# Use of artificial intelligence in oral radiology: a multicenter cross-sectional study in Egypt

**DOI:** 10.1186/s12903-025-07269-4

**Published:** 2025-11-25

**Authors:** Nora Saif, Ahmed Elkoumi, Abeer K. Shaalan

**Affiliations:** 1 Faculty of Dentistry, Cairo University, Giza, Egypt; 2 EMRA Lab (Egyptian Maxillofacial Radiology Alliance), Cairo, Egypt; 3 Faculty of Oral and Dental Medicine, Egyptian Russian University (ERU), Cairo, Badr City, Egypt; 4 Centre for Oral, Clinical & Translational Sciences, Faculty of Dentistry, Oral & Craniofacial Sciences, King’s College London, Guy’s Hospital, Tower Wing, London, UK

**Keywords:** Artificial intelligence, Oral radiology, Egyptian dentistry

## Abstract

**Background:**

AI-driven tools augment dentists’ capabilities by assisting to identify essential anatomical structures and improving the precision of diagnosing health conditions. However, the effective application of AI depends on dentists’ awareness and willingness to utilize these technologies proficiently. The Egyptian government is actively promoting the integration of AI into healthcare, yet there remains a notable gap in research concerning Egyptian dentists’ knowledge and attitudes towards AI in oral radiology. This study aimed to bridge this gap by assessing Egyptian dentists’ understanding of AI and identifying the barriers to its clinical implementation.

**Methods:**

A cross-sectional survey was conducted online among Egyptian dentists. The questionnaire assessed their knowledge, attitudes, and perceived challenges related to AI in oral radiology. Dentists were recruited through academic institutions, professional organizations, and social media platforms. To identify the factors influencing respondents’ knowledge of AI, a logistic regression analysis was applied.

**Results:**

Of the 399 participants, 50.3% reported being familiar with AI. Only 16.3% actively used AI in their clinical practice. A significant number (43.9%) learned about AI through self-study, whereas 14.8% attended conferences and workshops to gain knowledge. A strong majority (86%) supported the idea of integrating AI into dental education, 78% believed that AI could have a significant impact on oral radiology, and only 17% thought that AI could completely replace oral radiologists. This study identified several key barriers: limited knowledge (69.9%), lack of training opportunities (73%), and financial limitations (69.4%). The multivariable logistic regression analysis showed that attitudes toward AI were not significant predictors of AI knowledge. Participants working solely as practitioners had lower AI knowledge than those involved in policy or decision-making (OR = 0.44, 95% CI: 0.21–0.91, *p* = 0.026). Using multiple AI learning methods significantly improved knowledge compared to self-learning alone (OR = 3.72, 95% CI: 1.58–8.88, *p* = 0.008) and was more effective than any single method. Working in institutions with a clear AI implementation strategy was also associated with higher AI knowledge (OR = 3.31, 95% CI: 1.31–8.45, *p* = 0.012), while strategies still in development showed no significant benefit (AUC = 0.8).

**Conclusion:**

Egyptian dentists recognize the potential benefits of AI in oral radiology but face certain challenges, such as gaps in knowledge, training, and lack of strategic AI initiatives within their institutions.

**Trial registration:**

This study was registered at ClinicalTrials.gov with identifier NCT06908603.

**Supplementary Information:**

The online version contains supplementary material available at 10.1186/s12903-025-07269-4.

## Background

The integration of artificial intelligence (AI) has significantly transformed the field of dentistry, revolutionizing various aspects of diagnostic procedures, treatment planning, and clinical decision-making processes [1–3]. This transformation is largely driven by radiology, which serves as the primary gateway for AI integration into the medical field, with diagnostic images forming the core data source for developing AI systems [4].

Existing research on AI applications in dental and maxillofacial radiology span a wide range of clinical use cases, including automated identification of craniofacial anatomical structures and pathological conditions, classification of maxillofacial cysts and tumors, as well as diagnosis of dental caries and periodontal diseases [5].

Oral radiology, a cornerstone of modern dentistry, stands to benefit significantly from AI-driven tools. However, its successful implementation depends not only on technological advancements but also on the readiness of dental professionals to embrace this innovation [6].

The adoption of artificial intelligence (AI) in healthcare has rapidly increased globally [7–9]. In Egypt, the government has demonstrated a strong commitment to integrate AI into its healthcare system through the implementation of the National Artificial Intelligence Strategy, which emphasizes AI’s role in enhancing diagnostic capabilities and promoting public health [10]. The Egyptian Healthcare Authority (EHA) is actively exploring and implementing AI solutions to improve diagnostic precision and advance the digital health infrastructure [11] This national focus on AI in healthcare provides a supportive environment for the integration of AI into specialized fields, such as oral radiology. However, this requires a thorough understanding of the knowledge, perceptions, and preparedness of dental professionals. Despite the growing global and national interest in AI applications, there is limited research on the awareness and attitudes of Egyptian dentists toward AI in oral radiology and the barriers to its adoption in education, training, and clinical practice [12].

This multicenter cross-sectional investigation aimed to address the existing research gap by evaluating the knowledge and perceptions of Egyptian dentists regarding the application of artificial intelligence in dental imaging. By exploring their awareness of AI technologies, perceived benefits, and concerns, this study sought to provide valuable insights into the current state of AI readiness within the Egyptian dental community. Additionally, the survey aimed to identify the specific challenges associated with integrating AI into dental education and clinical practice, providing actionable recommendations for policymakers, educators, and practitioners in Egypt to harness the potential of AI to improve the delivery of oral healthcare.

## Materials and methods

This study was conducted according to the Strengthening the Reporting of Observational Studies in Epidemiology (STROBE) guidelines [13].

### Study design

This cross-sectional study assessed the knowledge and perceptions of Egyptian dentists regarding AI applications in dental imaging. This study also aimed to identify the needs and challenges of implementing this evolving technology in education, training, and clinical practice. The survey was designed using the DATAtab: Online Statistics Calculator (https://datatab.net/) and was active for participation from March to November 2024 with regular reminders.

### Questionnaire and variables

The online, self-administered 16-item questionnaire included several question styles (yes/no questions, closed-ended, multiple-choice questions, and statements with Likert-scale responses). The questionnaire was adapted from several published AI surveys [14–16]. The questions were designed to assess three primary areas: AI knowledge, perceptions of AI integration into practice, and challenges in implementing AI in OR practice in Egypt. As previous discussions with colleagues revealed that there was a lack of knowledge about the subject, we used a 3-point Likert-type scale to obtain a straightforward response from participants in a “forced-choice” response format [17] This was mainly done to encourage participants to carefully consider their responses and reduce the response bias that can occur when participants always select the neutral option. The questionnaire started with an overview of the aims of the study and an information leaflet highlighting confidentiality, voluntary participation, and the contact details of the author if further clarification is required. The privacy of all collected data was preserved, and the data was securely stored and accessible only to the research team.

### Pilot

Five dentists working in clinical and academic settings piloted the survey to assess its completion time, participants’ comprehension of the questions, validity, and reliability of the information. Some of the reviewers’ recommendations and comments have been used to rephrase the questions for clarity.

### Bias

To minimize potential bias, this study employed a diverse recruitment strategy, reaching participants through academic institutions, professional organizations, and social media platforms. However, selection bias may have been present because of the voluntary nature of participation, as dentists with a prior interest in AI may have been more likely to respond. Additionally, self-reported data may introduce recall or social desirability bias. Efforts were made to mitigate these biases by ensuring anonymity, using a standardized questionnaire, and objectively analyzing responses. In addition, data from the pilot study was excluded from the final analysis reported in this manuscript. Despite these measures, it is important to acknowledge that some degree of sampling bias may still be present, potentially limiting the generalizability of the findings to the broader population of dentists.

### Setting and participants

This cross-sectional study employed convenience and snowball sampling to recruit participants. The researchers engaged faculty members and postgraduate dental students at the Faculty of Dentistry, Cairo University, and members of the Egyptian Maxillofacial Radiology Alliance from various academic dental institutions across Egypt, including Alexandria University, Ain Shams University, Al-Azhar University, Beni-Suef University, Misr International University, Future University in Egypt, Misr University for Science and Technology, and the British University in Egypt. Additionally, social media platforms have been used to engage members of other Egyptian dental societies. The study included dentists with a bachelor’s or higher degree from Egyptian universities who practiced in Egypt. Undergraduate students and dentists who obtained undergraduate qualifications abroad or practiced outside Egypt were excluded.

### Sample size

In 2023, the Egyptian Dental Syndicate reported 76,843 members [18]. Consequently, we determined our sample size to represent this population, employing a 95% confidence level and 5% margin of error, which necessitated a minimum sample size of 382. The data was collected from 399 participants. The sample size was calculated using the Epi Info™ 7.2 (Centers for Disease Control and Prevention (CDC), Atlanta, USA).

### Ethical approval

This study was conducted in accordance with the Code of Ethics of the World Medical Association (Declaration of Helsinki) and approved by the Cairo University Faculty of Dentistry Ethics Committee (approval number 50-3-25).

### Outcomes

This study assessed the Egyptian dentists’ knowledge, attitudes, and perceptions regarding the application of AI in oral radiology. AI knowledge was measured based on how participants described their familiarity with artificial intelligence in dentistry. Those who answered *“Understand and practice AI”* were classified as having good knowledge, as they showed both understanding and practical use. Participants who chose *“Familiar but not enough to apply it in practice*,*” “Only basics I knew from media*,*”* or *“I have no idea”* were grouped as having poor knowledge, since their understanding was limited or only general. This grouping helped us clearly separate those with hands-on experience from those with only basic or no knowledge. The findings provide valuable insights into their level of awareness, readiness for AI adoption, and the challenges they face when integrating AI into clinical practice.

### Statistical methods

Data was collected using DATAtab and analyzed using Jamovi (2.6.13) and RStudio (2023.06.0 Build 421) software. Descriptive statistics, in the form of numbers and percentages, were used to present the demographic characteristics of the participants. Logistic regression analysis was conducted to determine the prognostic factors using both univariate and multivariate approaches. The following packages were used in the analysis: “glm” package for model development, “rms” package to generate calibration curves, “rmda” package for decision curve analysis, and “pROC” package to construct ROC curves and calculate the AUC. Statistical significance was set at *P* < 0.05.

To investigate determinants influencing dentists’ comprehension of artificial intelligence (AI), a logistic regression model was utilized and validated for accuracy and effectiveness. The analysis incorporated univariate and multivariate logistic regression to identify prognostic factors. The dataset was divided into training and test sets in an 8:2 ratio to construct the model, examining determinants of participants’ AI knowledge in dentistry. The outcome variable differentiated between high and low levels of AI knowledge. Predictor variables included age, gender, qualifications, specialty, work environment, experience, self-perception, attitude towards AI, AI skill development, and presence of an AI implementation strategy in the workplace.

The logistic regression model provides odds ratios for each predictor. Variables with insufficient participant representation were excluded. The model’s accuracy and predictability were evaluated using the area under the curve (AUC), receiver operating characteristic (ROC) curves, and calibration plots. The model was validated on the test set using its ROC curve, AUC, and calibration curve. A decision curve analysis was conducted to verify the model’s generalizability and applicability to broader populations and other dental professionals. This approach identified key factors associated with AI knowledge among dentists and confirmed that our predictive models are statistically robust and suitable for practical application.

## Results

### Participants’ demographics

**Fig. 1:**
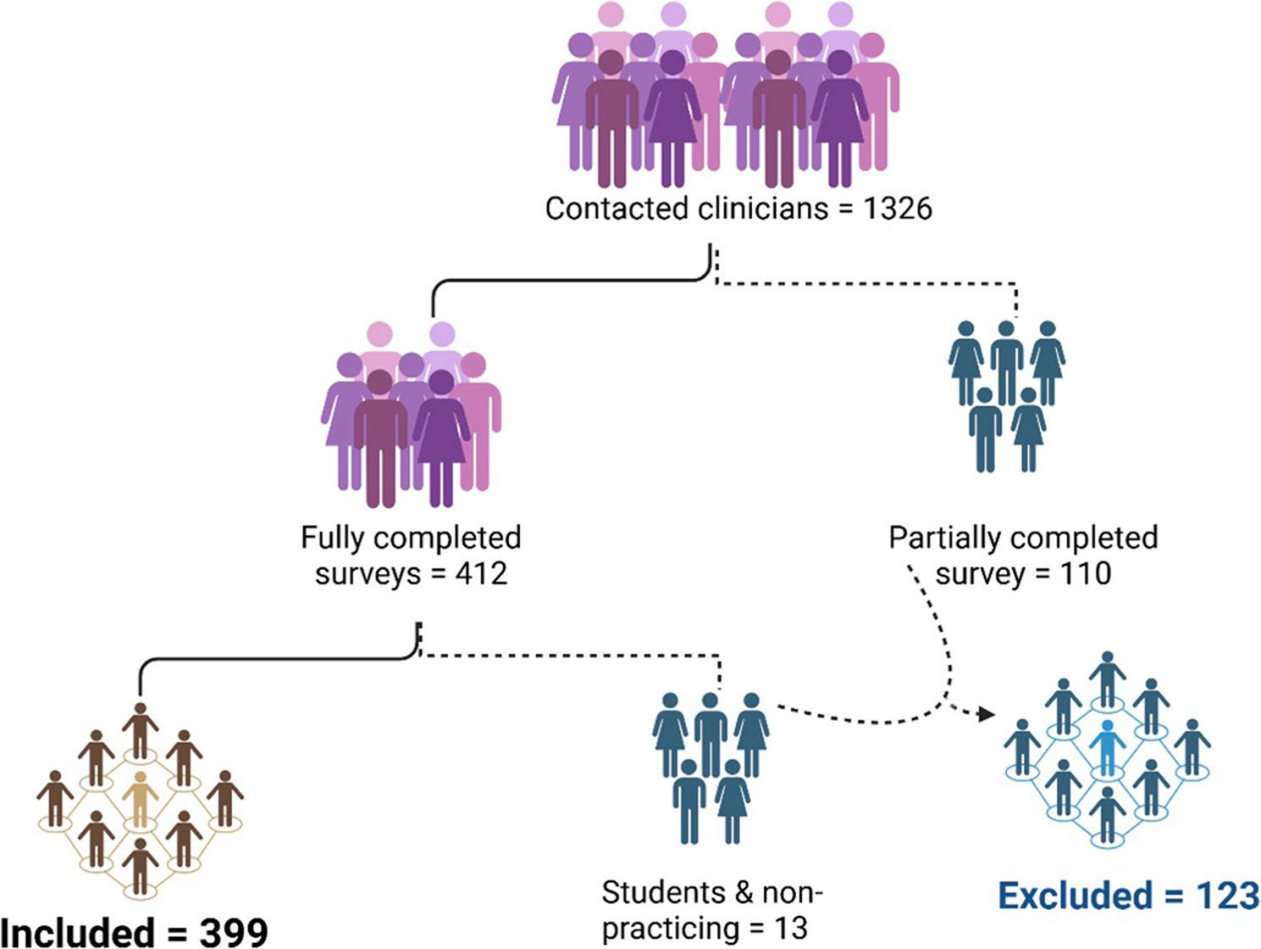
Participants’ selection process

A total of 1,326 clinicians were invited to participate in the survey. Among them, 412 fully completed the survey, while 110 provided only partial responses. After applying the eligibility criteria, 13 responses from students or non-practicing dentists were excluded, along with 110 partially completed surveys. In total, 399 respondents were included in the final analysis (Fig. 1).

Table 1 demonstrates that the majority of participants (52.6%) were aged between 30 and 49 years, while 38.8% were under 30 years, and 8.5% were over 50 years. The sample predominantly comprised females (65.9%), with males constituting 34.1%. Regarding educational qualifications, 37.3% held a Bachelor of Science (BSc) degree, 25.3% a Master of Science (MSc) degree, and 37.3% a Doctor of Philosophy (PhD) degree. Clinical experience varied among respondents, with 35.1% having 0–5 years of practice, 15.3% having 6–10 years, 13.5% having 11–15 years, and 36.1% possessing over 15 years of experience. The most prevalent workplace was academia (45.1%), followed by private practice (27.1%) and national health institutes (10.8%), while 17% were employed across multiple settings. In terms of specialization, 35.1% were identified as oral radiologists, whereas 64.9% belonged to other specialties. Concerning authority, 48.4% reported being decision-makers with policy implementation responsibilities, whereas 51.6% identified themselves as practitioners only.

**Table 1:** Demographic characteristics of the participants

	*N* (%)
**Age Range**	
<30	155 (38.8%)
30–49	210 (52.6%)
>50	34 (8.5%)
**Gender**	
Female	263 (65.9%)
Male	136 (34.1%)
**Qualification**	
BSc	149 (37.3%)
MSc	101 (25.3%)
PhD	149 (37.3%)
**Years of Practice**	
0–5 yrs	140 (35.1%)
6–10 yrs	61 (15.3%)
11–15 yrs	54 (13.5%)
>15	144 (36.1%)
**Current workplace**	
Academia	180 (45.1%)
Private Practice	108 (27.1%)
National Health Institute	43 (10.8%)
More than a work setting	68 (17)
**Specialty**	
Oral radiologist	140 (35.1%)
Other	259 (64.9%)
**Self-Description**	
I am decision maker who can implement policies	193 (48.4%)
I am practitioner only	206 (51.6%)

### Knowledge and perceptions of AI

The survey assessed participants’ knowledge, learning sources, perceptions, and workplace strategies regarding artificial intelligence (AI). When asked about their understanding of AI, half of the respondents (50.3%) reported being familiar with the concept, but not confident enough to apply it in practice. Meanwhile, 28.8% had only basic knowledge acquired from media, 16.3% understood AI and actively used it, while 4.5% had no knowledge at all. Regarding the sources of AI knowledge, 43.9% were self-taught, 14.8% had attended conferences or workshops, and 9% had completed formal AI education or training. Additionally, 3.3% gained knowledge from company sales representatives, whereas 16.3% acquired AI understanding through a combination of sources. Notably, 12.8% of the participants reported having no AI knowledge.

Participants’ attitudes toward AI varied, with 46.6% expressing excitement about its potential, 18.5% acknowledging its challenges, and 7.8% feeling worried about its impact. Additionally, 13.03% admitted that they did not know enough to form a clear opinion, while 14% reported a mixed perception that combined excitement, concerns, and awareness of challenges. Regarding AI strategies in their workplaces, 46.6% stated that no AI strategy was in place, whereas 19.3% reported that a strategy was under development. Furthermore, 19% were unaware of any AI plans, and only 15.04% confirmed that their organization had an AI strategy in place (Table 2)

**Table 2:** Knowledge of AI

		*N* (%)	
**How well do you understand what AI means?**			
Familiar but not enough to apply it in practice		201 (50.3%)	
Only basics I knew from media		115 (28.8%)	
Understand and practice AI		65 (16.3%)	
I have no idea		18 (4.5%)	
**How did you develop your AI knowledge?**			
Self-taught		175 (43.9%)	
Conferences/workshop		59 (14.8%)	
No knowledge		51 (12.8%)	
Completed AI education/training		36 (9.0%)	
From a company sales/representative		13 (3.3%)	
Combination of the above		65 (16.3%)	
**How do you feel about AI?**			
Excited		186 (46.6%)	
Aware of challenges		74 (18.5%)	
I don’t know enough		52 (13.03%)	
Worried about the impact		31 (7.8%)	
Mixed Feelings		56. (14%)	
**Does your workplace/organization have a strategy for AI?**			
No		186 (46.6%)	
A strategy is under development		77 (19.3%)	
I have no idea		76 (19%)	
Yes		60 (15.04%)	

**Fig. 2:**
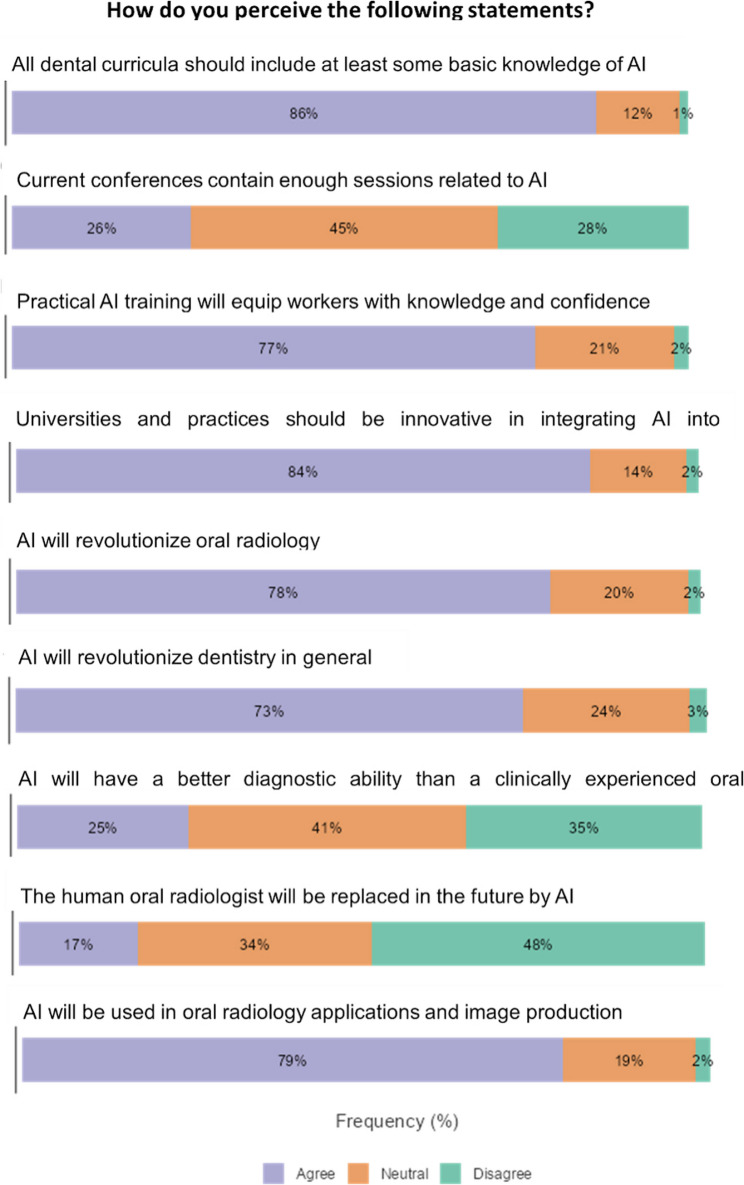
Participants’ perceptions of AI in dentistry and oral radiology

The survey further evaluated participants’ perceptions of artificial intelligence (AI) in the fields of dentistry and oral radiology, uncovering substantial support for its integration into educational curricula and professional practice (Fig. 2). A significant majority (86%) concurred that dental curricula should incorporate AI knowledge, while 77% believed that practical AI training would enhance their confidence and skills. Similarly, 84% endorsed innovation in AI adoption within universities and dental practices. Concerning AI’s impact, 78% anticipated it would revolutionize oral radiology, and 73% held similar expectations for dentistry as a whole. However, opinions on AI’s diagnostic superiority were varied, with 25% agreeing that AI would surpass clinically experienced radiologists, 41% remaining neutral, and 35% disagreeing. Skepticism increased regarding AI’s potential to replace human oral radiologists, with only 17% in agreement, 34% neutral, and 48% in disagreement. While 79% believed AI would be extensively utilized in oral radiology applications and imaging, perspectives on its presence in current conferences were divided, with 26% agreeing that sufficient AI-related sessions exist, 45% neutral, and 28% disagreeing. Overall, while there is optimism regarding AI’s role in dentistry, concerns persist about its ability to surpass human expertise and fully replace professionals in the field.

**Fig. 3:**
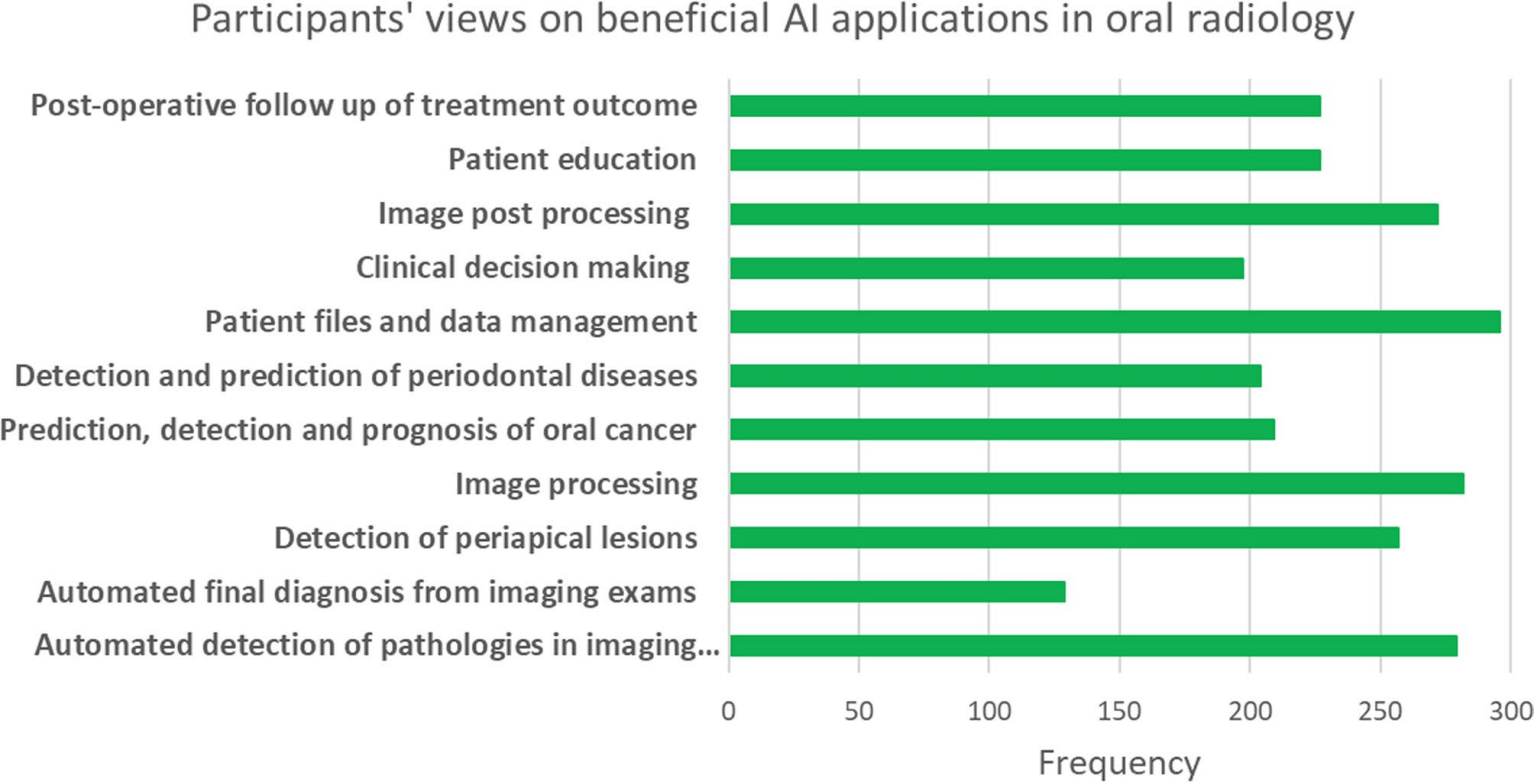
Participants’ perspectives on the most beneficial AI applications in oral radiology

The survey explored the participants’ perspectives on the most beneficial applications of AI in oral radiology (Fig. 3). The results indicate that patient files and data management received strong support as key applications, reflecting AI’s potential to enhance efficiency in handling patient records. Image post-processing, including image enhancement and artifact reduction, has also been widely recognized as beneficial, similar to image processing for cephalometric tracing and tooth segmentation. AI’s role in automated detection of pathologies in imaging examinations was another highly endorsed application, alongside its use in detecting and predicting periodontal diseases and periapical lesions, suggesting that participants see AI as a valuable diagnostic aid. Additionally, AI has been considered valuable in clinical decision-making, such as in selecting and positioning imaging modalities, as well as in the prediction, detection, and prognosis of oral cancer. While patient education and post-operative follow-up of treatment outcomes were also considered useful, AI’s role in automated final diagnosis from imaging exams received comparatively lower endorsement, indicating that participants may still rely on human expertise for final clinical decisions. Overall, these findings highlight a strong belief regarding the role of AI in enhancing diagnostic accuracy, streamlining workflow, and improving image analysis in oral radiology.

### Barriers to AI integration in dental practice

The survey investigated the primary obstacles impeding the integration of artificial intelligence (AI) into dental practice and institutions (Table 3). A lack of knowledge was identified as a significant barrier, with 69.9% of respondents concurring that insufficient understanding of AI hinders its adoption, while 23% remained neutral and only 7% disagreed. Similarly, skill development was recognized as a substantial challenge, with 66.9% agreeing that a deficiency in AI-related skills obstructs implementation, 29.8% neutral, and 3.4% disagreeing. The availability of educational and training courses was another critical factor, as 73% agreed that limited access to AI training constitutes a barrier, whereas 21.3% remained neutral and 5.6% disagreed. The difficulty of implementing AI in daily practice elicited more divided opinions, with 40.4% agreeing that integration is challenging, 46.3% neutral, and 13.2% disagreeing, suggesting that some practitioners may perceive AI as feasible under certain conditions. Financial cost was another major concern, with 69.4% agreeing that the expense of AI adoption is a limiting factor, while 25.3% were neutral, and only 5.3% disagreed. These findings indicate that while AI holds promise in dentistry, barriers related to knowledge, training, skills, financial costs, and practical feasibility must be addressed to facilitate widespread integration.

**Table 3:** Obstacles hindering the integration of AI in dental practice and institutions

	Agree *N*(%)	Neutral *N*(%)	Disagree *N*(%)
Knowledge	249 (69.9%)	82 (23.0%)	25 (7.0%)
Skill development	238 (66.9%)	106 (29.8%)	12 (3.4%)
Availability of education & training courses	260 (73.0%)	76 (21.3%)	20 (5.6%)
Hard to implement in work & practice	144 (40.4%)	165 (46.3%)	47 (13.2%)
Financial cost	247 (69.4%)	90 (25.3%)	19 (5.3%)

The survey also examined the perceived importance of various challenges associated with the implementation of artificial intelligence (AI) in radiology (Table 4). The integration of information technology and computer science into radiology education and curricula emerged as a primary concern, with 50.7% of respondents considering it very significant, 46.4% significant, and only 2.9% non-significant, underscoring the necessity for enhanced AI education. Similarly, the collaboration among educators, administrators, and radiologists was deemed essential, with 48.3% rating it very significant, 48.5% significant, and only 3.2% non-significant, indicating a strong consensus on the importance of interdisciplinary cooperation. The presence of leaders and decision-makers capable of advancing AI applications was identified as the most critical factor, with 57.5% considering it very significant, 40.1% significant, and only 2.4% non-significant, highlighting the need for robust leadership in AI adoption. The absence of regulatory policies governing AI use was another notable concern, with 45.6% viewing it as very significant, 47.5% as significant, and 6.9% as non-significant, reflecting uncertainty regarding ethical and legal frameworks. Lastly, the lack of assigned responsibility for AI-based decisions was perceived as a key issue, with 47.8% rating it very significant, 45.9% significant, and 6.3% non-significant, suggesting apprehension about accountability in AI-driven clinical decisions. These findings indicate that education, collaboration, leadership, regulatory frameworks, and accountability are critical challenges that must be addressed for the successful implementation of AI in radiology.

**Table 4:** Challenges related to AI implementation in radiology

	Very Significant *N* (%)	Significant *N* (%)	Non-significant *N* (%)
1. Integration of IT and computer science in radiology education and curriculum	192 (50.7%)	176 (46.4%)	11 (2.9%)
2. Collaboration between educators, administrators and radiologists	183 (48.3%)	184 (48.5%)	12 (3.2%)
3. Presence of leaders and decision makers who can transform AI applications in radiology work	218 (57.5%)	152 (40.1%)	9 (2.4%)
4. The lack of regulatory policy which governs AI use	173 (45.6%)	180 (47.5%)	26 (6.9%)
5. The lack of assigned responsibility for consequences of AI-based decisions	181 (47.8)	174 (45.9%)	24 (6.3%)

### Factors influencing AI knowledge

**Fig. 4:**
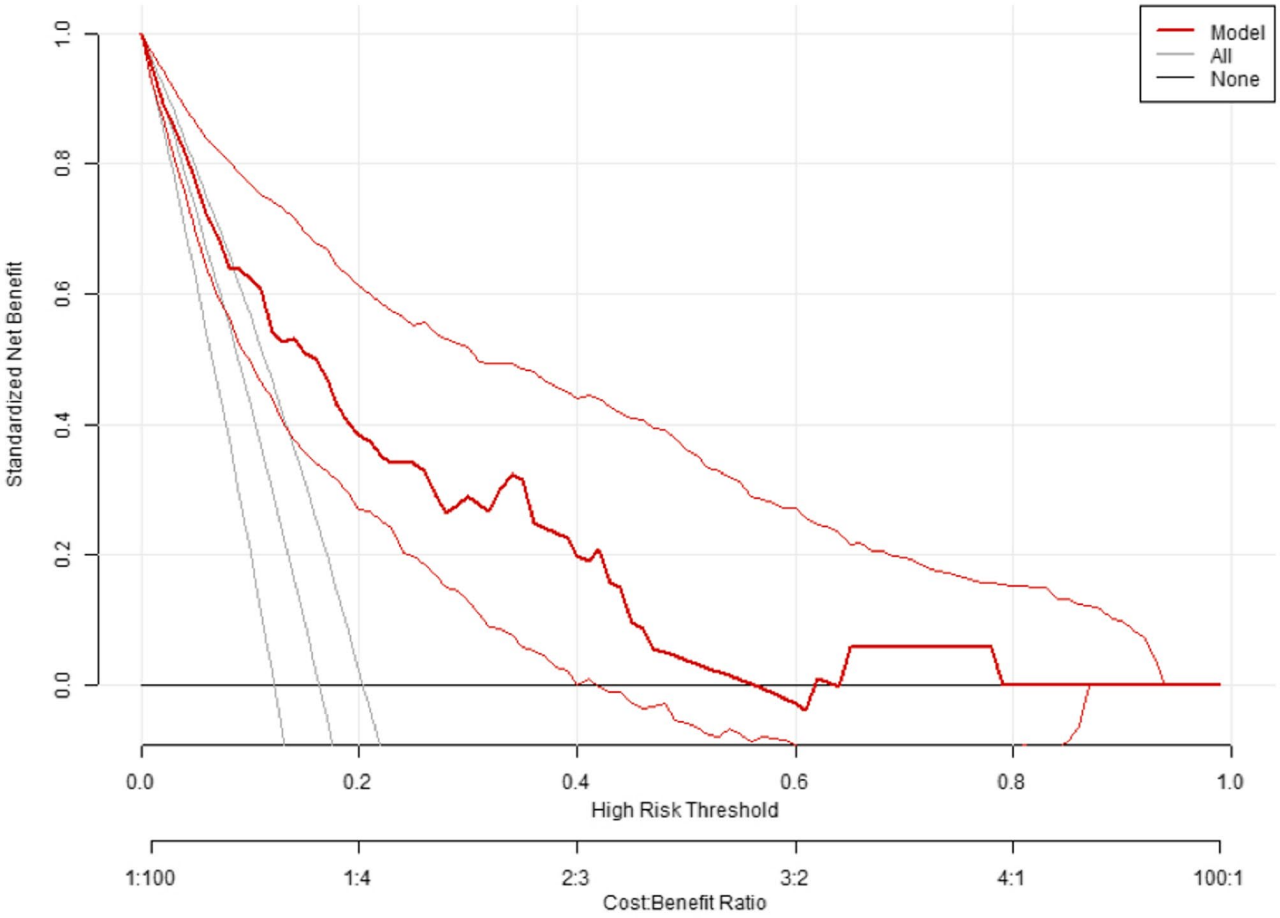
Decision Curve Analysis (DCA) shows the net benefit of the logistic regression model created across a range of high-risk thresholds (x-axis). The y-axis represents the standardized net benefit, where higher values indicate greater benefits and applicability

A logistic regression model was developed using the training data to identify factors influencing participants’ AI knowledge, and the test set was used to evaluate the model’s accuracy and discrimination ability. The logistic regression model yields clear insights, with each predictor’s impact quantified by odds ratios, thus facilitating an easier interpretation of the data. Our dataset was divided into training (80%) and test (20%) sets with 319 and 80 participants in the training and test sets, respectively. The model achieved an AUC of 0.803, indicating strong discriminatory power (Supplementary Fig. 1). The calibration plot demonstrates a high degree of agreement between the predicted and observed values [Supplementary Fig. 2]. To assess the practicality and generalizability of the model, a decision curve analysis was conducted, confirming its reliability and applicability in practice (Fig. 4). These findings provide valuable insights for future research and practical strategies to improve dentists’ awareness and understanding of AI in dentistry.

Univariate analysis examined several factors, including age, gender, qualification, specialty, work setting, experience, self-description, feelings about AI, AI learning methods and whether the workplace had an AI strategy. This revealed that self-description, AI learning methods, and the presence of an AI strategy in the workplace were statistically significant factors affecting participants’ AI knowledge. However, in the multivariate model, feelings about AI did not remain significant. Participants who identified solely as practitioners had significantly lower AI knowledge than those involved in decision-making and policy implementation (OR: 0.44, 95% CI [0.21, 0.91], p = 0.026). Using multiple sources or a combination of AI learning methods significantly improved AI knowledge compared to self-learning alone (OR: 3.72, 95% CI [1.58, 8.88], p = 0.008) and was more effective than any single learning source. Additionally, working in an environment with a clear AI implementation strategy significantly enhanced AI knowledge compared with workplaces without such a strategy (OR: 3.31, 95% CI [1.31, 8.45], p = 0.012). Notably, participants in workplaces with an AI strategy that was still under development did not show significantly better AI knowledge than those in workplaces without any strategy (Table 5).

**Table 5:** Univariable and multivariable logistic regressions were used to detect factors influencing participants’ knowledge of AI

Characteristic	Univariable			Multivariable		
	OR ^1^	95% CI ^1^	*p*-value	OR ^1^	95% CI ^1^	*p*-value
**Age**			>0.9			
< 30	—	—				
30–49	0.99	0.53, 1.88				
>50	0.97	0.26, 2.90				
**Gender**			0.8			
Male	—	—				
Female	1.07	0.57, 2.08				
**Qualification** *(Highest)*			0.4			
BSc	—	—				
MSc	1.24	0.55, 2.74				
PhD	1.57	0.79, 3.20				
**Specialty**			0.2			
GP	—	—				
Oral radiologist	1.86	0.86, 4.23				
Specialist (other)	2.00	0.93, 4.52				
**Experience**			0.064			
0–5 yrs	—	—				
6–10 yrs	1.96	0.81, 4.71				
11–15 yrs	0.50	0.11, 1.62				
>15	1.75	0.86, 3.67				
**Work setting**			0.2			
Academia	—	—				
National Health Institute	0.43	0.10, 1.33				
Private Practice	0.84	0.39, 1.75				
Multiple work settings	1.68	0.77, 3.58				
**Self-description**			< 0.001			0.026
I am decision-maker who can implement policies	—	—		—	—	
I am a practitioner only	0.33	0.17, 0.61		0.44	0.21, 0.91	
**Development of AI knowledge**			< 0.001			0.008
Self-taught	—	—		—	—	
Completed AI education/training	2.63	0.97, 6.70		1.76	0.58, 5.03	
Conferences/workshop	0.77	0.21, 2.24		0.63	0.16, 1.97	
From company sales/representative	1.97	0.28, 8.74		1.65	0.19, 9.65	
No knowledge	0.21	0.01, 1.10		0.31	0.02, 1.70	
Combination of methods	5.33	2.51, 11.6		3.72	1.58, 8.88	
**Feelings about AI**			0.008			0.3
Excited	—	—		—	—	
Aware of challenges	1.05	0.47, 2.23		1.33	0.55, 3.12	
Worried about the impact	0.44	0.07, 1.63		0.92	0.13, 3.84	
I don’t know enough	0.14	0.01, 0.71		0.30	0.02, 1.69	
Mixed Feelings	2.15	0.95, 4.74		1.97	0.76, 4.98	
**AI strategy in the work setting**			< 0.001			0.012
No	—	—		—	—	
I have no idea	0.57	0.16, 1.63		0.50	0.13, 1.55	
A strategy is under development	2.59	1.14, 5.81		1.44	0.58, 3.51	
Yes	6.03	2.77, 13.4		3.31	1.31, 8.45	

^1^*OR* Odds Ratio, *CI* Confidence Interval

## Discussion

Artificial intelligence (AI) is increasingly reshaping radiology, medicine, and healthcare, marking radiology as a pioneering specialty in AI adoption [19]. AI applications in oral radiology have shown promise in areas such as image analysis, lesion detection, and treatment planning [20]. The integration of AI in oral radiology has the potential to enhance diagnostic accuracy, improve workflow efficiency, and assist in complex case interpretations [5].

Dentists’ perspectives on artificial intelligence (AI) are crucial for its successful integration into oral radiology and dental healthcare. Studies have shown that dental professionals generally have a positive attitude towards AI and recognize its potential benefits in improving diagnostic accuracy and efficiency [21, 22] This study investigated dentists’ attitudes, knowledge, and expectations concerning AI in their professional practice, particularly in the field of oral radiology. Although previous research has examined the perceptions of dental and medical students regarding AI, this study is the first to specifically focus on practicing dentists in Egypt and their views on AI in oral radiological diagnostics and applications [23, 24].

The diverse cohort in this study provided comprehensive insights into the perception of artificial intelligence (AI) within the field of dentistry. While 50.3% of respondents were familiar with AI concepts, they lacked confidence in applying them practically. Aldakhil et al. [25] reported similar awareness levels (50.31%), and Ourang et al. [26] highlighted endodontists’ limited understanding of machine learning principles. This gap between theoretical knowledge and practical implementation is especially pronounced in low- and middle-income countries, as noted by Umer et al. [27]. The significant proportion of self-taught AI knowledge (43.9%) underscores the need for formal AI training in dental education.

The survey findings reveal a notable degree of enthusiasm and optimism concerning the potential applications of artificial intelligence (AI) in dentistry and oral radiology. The overwhelming agreement that AI will revolutionize oral radiology and dentistry, shared by 78–84% of participants, suggests widespread recognition of the profound impact AI is expected to have on diagnostic procedures, treatment planning, and overall patient care in these fields. Approximately half of the participants expressed excitement about AI’s future prospects, indicating a rising trend of interest and acceptance within the dental community. This trend aligns with observations from other global multicentric studies [28]. The strong support for integrating AI into dental curricula, with 86% of respondents agreeing on its importance, highlights the perceived need for future dental professionals to be well-versed in AI technologies. As AI continues to advance and find applications in various aspects of dental practice, there is a growing consensus that education and training in AI-related concepts should be incorporated into dental education to prepare students for the evolving landscape of their profession [14, 23, 28, 29].

The mixed opinions on AI’s diagnostic capabilities in radiology reflect the complex and evolving landscape of artificial intelligence in healthcare. While approximately 25% of respondents expressed confidence in the AI’s potential to exceed the proficiency of seasoned radiologists, a more substantial segment remained skeptical or uncertain. The division within the medical community regarding the role and effectiveness of artificial intelligence (AI) in diagnostic procedures is evident [30–32]. Skepticism may stem from concerns about AI’s capacity to accurately interpret complex or atypical cases, as well as the value of human intuition and experience in clinical decision-making. Such reservations may further reflect ethical concerns about depending exclusively on AI for critical medical decisions and emphasize the significance of human interaction in patient care. Despite technological progress in AI, these perspectives indicate that many experts view AI as a supportive tool rather than a replacement for human expertise in oral radiology [33–35]. This spectrum of perspectives underscores the importance of ongoing dialogue and research to address the promises and challenges of implementing AI in oral radiology.

The identification of key areas where AI could be most beneficial demonstrates a balanced understanding of the strengths and limitations of the technology within the field. Strong support for AI in image processing, automated pathology detection, and clinical decision-making suggests that professionals recognize the potential of AI to enhance diagnostic accuracy and efficiency. However, the emphasis on patient files and data management as a primary application indicates that AI’s role in streamlining administrative tasks is equally valued. This balanced perspective emphasizes the diverse potential of artificial intelligence in oral radiology, extending to both clinical practice and healthcare operations.

The survey identified several significant barriers to AI integration into dental practice. Lack of knowledge (69.9%) and limited access to AI training (73%) emerged as primary obstacles, highlighting the urgent need for comprehensive AI education and training programs in dentistry. Similarly, in their international survey on AI in radiology, Huisman et al. identified lack of knowledge as a major bottleneck for AI implementation in the radiological community [36]. The financial cost of AI adoption (69.4% agreed) was also identified as a major barrier, suggesting that economic factors play a crucial role in the slow integration of AI technologies. Several studies have identified the financial burden of adopting AI technologies as a significant barrier, particulary in resource-poor health institutions and in low and middlee income countires (LMIC). This includes the costs associated with acquiring the necessary equipment, software, and infrastructure, as well as the ongoing expenses for maintenance and updates [27, 37, 38]. Many LMICs face challenges in securing adequate funding for AI initiatives in healthcare, which further impedes the adoption process [39, 40]. More research is needed to fully understand the economic impact of AI in dentistry, particularly in low and middle-income countries where resources may be more limited [27].

The challenges related to AI implementation in radiology further emphasize the need for a multifaceted approach to AI integration. The high importance placed on leadership (57.5% considered very significant) and collaboration among educators, administrators, and radiologists (96.8% considered significant or very significant) underscores the need for coordinated efforts to drive AI adoption in the field. Kim et al. stressed the importance of a holistic approach that addresses challenges spanning technology, workflow, and organizational levels [41]. Meanwhile, Yang et al. pointed out that numerous stakeholders have indicated the need for cooperation between radiologists and AI experts to effectively enhance patient care [42].

The logistic regression model revealed several key factors that influence AI knowledge among dental professionals. Notably, practitioners involved in decision making and policy implementation demonstrated higher AI knowledge than those involved solely in clinical practice. This finding suggests that engagement with broader aspects of dental practice may facilitate greater exposure to and understanding of AI technology. Additionally, the positive impact of working in an environment with a clear AI implementation strategy emphasizes the role of institutional support in fostering AI knowledge and adoption. The effectiveness of multiple learning sources in improving AI knowledge highlights the importance of diverse and comprehensive AI education strategies. This approach is particularly relevant given AI’s significant potential to reshape dental education and practice [43, 44].

Finally, the developed logistic regression model, with its robust discriminatory power and practical applicability, can serve as an effective tool for assessing and predicting AI knowledge levels among dental professionals. These findings can inform the development of targeted educational programs and policies to enhance AI literacy in the dental field, ultimately improving patient care and advancing the profession.

Several limitations in our study should be acknowledged. Firstly, although anonymous participation was ensured, a standardized questionnaire was employed, and objective statistical methods were used, certain biases, such as social desirability and individual subjectivity, could not be entirely eliminated. This limitation is inherent in studies relying on self-reported data. Furthermore, as the sample was drawn from a specific geographic region, the generalizability of the findings may be limited. Nevertheless, we conducted a comprehensive model evaluation using the AUC curve, calibration plot, and decision curve analysis to enhance the reliability and potential applicability of our results. Additionally, the classification of AI knowledge into “good” and “poor” was based on self-reported responses to a single multiple-choice question. While this approach provided a practical means to distinguish between basic awareness and practical understanding, the cutoff remains somewhat arbitrary and has not been externally validated. This binary classification allowed for meaningful analysis using logistic regression, although future studies should consider validating or refining this measure using standardized or multi-item scales. Lastly, as the data were collected through an online survey, individuals without reliable internet access or adequate digital literacy may have been underrepresented, potentially introducing selection bias.

## Conclusion

The survey captures the intricate dynamics shaping AI acceptance in dental and radiological workflows. While there is recognition of AI’s potential, barriers remain in knowledge, training, and implementation. These findings underscore the need for AI education in dental curricula, training programs, and strategies to support adoption. Future efforts should focus on bridging theoretical understanding and practical application of AI in dentistry, addressing financial barriers, and fostering collaboration to drive AI integration. The regression model can identify groups less likely to possess adequate AI knowledge, informing targeted training programs and policies aimed at enhancing AI utilization across the dental sector.

## Supplementary Information

Supplementary Material 1

## Data Availability

All data generated or analyzed during this study are included in this published article and its supplementary information files.
