# Supplementary material for: Use of artificial intelligence in oral radiology: a multicenter cross-sectional study in Egypt
Source: BMC Oral Health. 2025 Nov 25;25:1888. doi: 10.1186/s12903-025-07269-4 (PMC12687518; doi:10.1186/s12903-025-07269-4)
Supplement: Supplementary file 1 — Supplementary Material 1 [file 12903_2025_7269_MOESM1_ESM.docx]

# **Use of Artificial Intelligence in Oral Radiology: A Multicenter Cross-Sectional Study in Egypt**

Nora Saif ^1,2*^, Ahmed Elkoumi ^3^ and Abeer K Shaalan ^4^

^1^ Faculty of Dentistry, Cairo University, Egypt

^2^ Lead researcher, EMRA Lab (Egyptian Maxillofacial Radiology Alliance)

^3^ Faculty of Oral and Dental Medicine, Egyptian Russian University (ERU), Cairo, Badr City, Egypt

^4^ Centre for Oral, Clinical & Translational Sciences, Faculty of Dentistry, Oral & Craniofacial Sciences, King’s College London, Guy’s Hospital, Tower Wing, London, United Kingdom

^*^ Corresponding author: Nora Saif ([nora.taha@dentistry.cu.edu.eg](mailto:nora.taha@dentistry.cu.edu.eg))


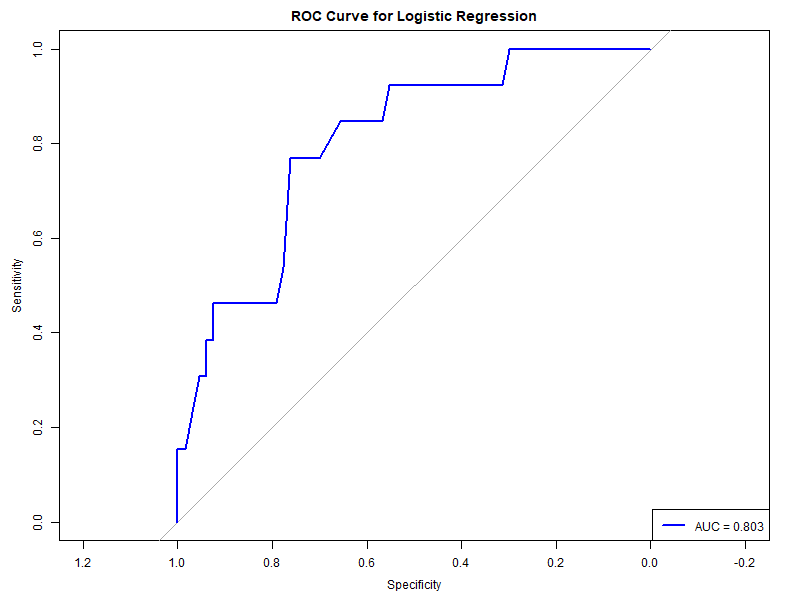


**Supplementary Fig. 1.** ROC curve of the multivariable logistic regression model illustrating the potential factors that may impact AI knowledge among dentists.


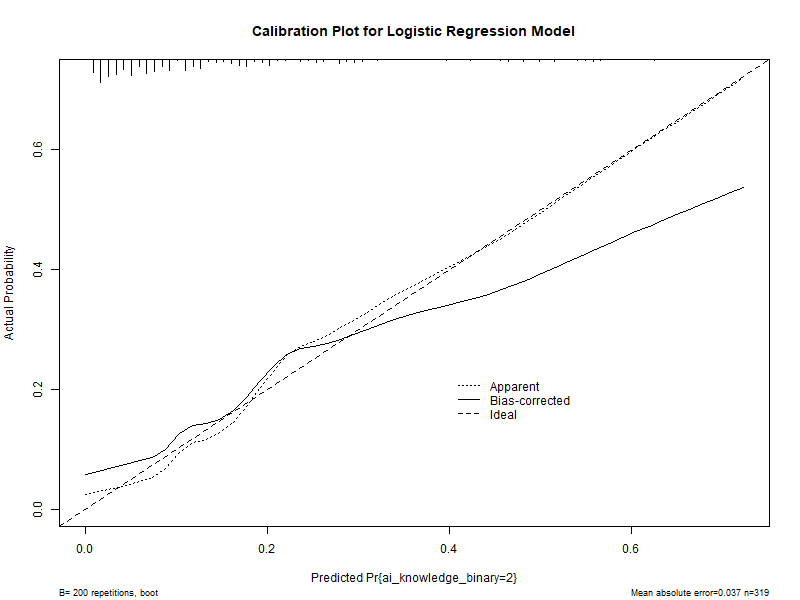


**Supplementary Fig. 2.** Calibration plot of the multivariable logistic regression model demonstrating a strong agreement between predicted and observed values.

#### **Use of Artificial Intelligence in Oral Radiology: A Multicenter Cross-Sectional Study in Egypt**

###### The survey aims to assess knowledge and perceptions of AI applications in dental imaging among Egyptian dentists. It also aims to identify the needs and challenges of implementing this transformative technology into education, training, and clinical practice.

##### **Information Leaflet**

###### The questionnaire will take approximately 5-10 minutes to complete. Confidentiality: All data will be collected anonymously with no identifiable markers of the participants. Information will be kept confidential and stored on a secure server. Participation and withdrawal: Contribution to the service evaluation is voluntary and you may decline to participate without consequence. Once the questionnaire has been completed, however, you will be unable to withdraw from the study. Contact details: If you have any questions or if you require any further information, please contact: Dr Nora Saif: [nora.taha@dentistry.cu.edu.eg](mailto:nora.taha@dentistry.cu.edu.eg)

##### **Demographics**

##### What is your age range?

- - <30
  - 30-49
  - >50

##### What is your gender?

- - Male
  - Female

##### What is your highest qualification?

- - BSc
  - MSc
  - PhD

##### Experience/years of practice

- - 0-5 yrs
  - 6-10 yrs
  - 11-15 yrs
  - >15

##### I am currently primarily working in

- - Private practice
  - Academia
  - National health institute

##### What is your field of speciality?

- - GP
  - Specialist (oral radiologist)
  - Specialist (other)
  - Other

##### How do you describe yourself?

- - I am a practitioner only
  - I am a decision maker who can implement policies

##### **Respondents' Knowledge of Artificial Intelligence (AI)**

##### How do you feel about AI?

- - Excited
  - Aware of challenges
  - Worried about the impact
  - I don't know enough
  - Neutral

##### How well do you understand what AI means?

- - I have no idea
  - Only basics I knew from media
  - Familiar but not enough to apply it in practice
  - Understand and practice AI

##### How did you develop your AI knowledge?

- - Self-taught
  - Completed AI education/training
  - Conferences/workshop
  - No knowledge
  - From company sales/representatives

##### **AI knowledge and education**

|  | Agree | Neutral | Disagree |
| --- | --- | --- | --- |
| All dental curricula should include at least some basic knowledge of AI |  |  |  |
| Current conferences contain enough sessions related to AI |  |  |  |
| Practical AI training will equip workers with knowledge and confidence |  |  |  |
| Universities and practices should be innovative in integrating AI into education |  |  |  |

##### **Perceptions of AI integration into practice**

##### How do you perceive the following statements:

|  | Agree | Neutral | Disagree |
| --- | --- | --- | --- |
| AI will revolutionize oral radiology |  |  |  |
| AI will revolutionize dentistry in general |  |  |  |
| AI will have a better diagnostic ability than a clinically experienced oral radiologist |  |  |  |
| The human oral radiologist will be replaced in the future by AI |  |  |  |
| AI will be used in oral radiology applications and image production such as quality control, dose selection, and image interpretation |  |  |  |

##### Does your workplace/organization have a strategy for AI?

- - YES
  - NO
  - I have no idea
  - A strategy is under development

##### **AI practice and challenges**

##### What AI applications do you believe would be beneficial for your dental practice/specialty?

- - Automated detection of pathologies in imaging exams
  - Automated final diagnosis from imaging exams
  - Detection of periapical lesions
  - Image processing (e.g. cephalometric tracing, tooth numbering, delineation of anatomical structures)
  - Prediction, detection and prognosis of oral cancer
  - Detection and prediction of periodontal diseases
  - Patient files and data management
  - Clinical decision making (e.g. selection and positioning of dental implant, risk evaluation for third molar extraction)
  - Image post processing (e.g. image enhancement, artifact reduction)
  - Patient education
  - Post-operative follow up of treatment outcome

##### Which of the following factors do you believe are hindering the integration of AI in your practice/institution?

|  | Agree | Neutral | Disagree |
| --- | --- | --- | --- |
| Knowledge |  |  |  |
| Skill development |  |  |  |
| Availability of education and training courses in AI |  |  |  |
| Hard to implement in work and practice |  |  |  |
| Financial cost |  |  |  |

##### The following are considered challenges regarding AI implementation. How do you view the significance of these challenges?

|  | Very significant | Significant | Non significant |
| --- | --- | --- | --- |
| Integration of IT and computer science in radiology education and curriculum |  |  |  |
| Collaboration between educators, administrators and radiologists |  |  |  |
| Presence of leaders and decision makers who can transform AI applications in radiology work |  |  |  |
| The lack of regulatory policy which governs AI use |  |  |  |
| The lack of assigned responsibility for consequences of AI-based decisions |  |  |  |
